# Treatment Outcomes of Temporomandibular Disorders Using Stabilization Splint Supported by the T-scan System

**DOI:** 10.1055/s-0045-1809915

**Published:** 2025-07-15

**Authors:** Hoang Kim Loan, Hoang Viet Hai, Nguyen Anh Tung, Nguyen Manh Phu, Nguyen Minh Duc

**Affiliations:** 1 School of Dentistry, Hanoi Medical University, Dong Da, Hanoi, Vietnam

**Keywords:** temporomandibular disorders, stabilization splints, T-scan system, pain reduction

## Abstract

**Objective:**

This study aims to evaluate the treatment outcomes of temporomandibular disorders (TMD) using stabilization splints (SS) supported by the T-scan system at Hanoi Medical University Hospital.

**Materials and Methods:**

A clinical, non-controlled intervention study was conducted from May 2023 to October 2024. A total of 36 patients (7 male, 29 female) diagnosed with TMD according to the Diagnostic Criteria for Temporomandibular Disorders (DC/TMD) were included. All patients received treatment with stabilization splints, and the T-scan system (Tekscan, Norwood, MA, USA) was employed to assist in adjusting occlusal contacts and force distribution of the SS. Clinical outcomes, including pain scores (visual analog scale, VAS), mouth opening range (maximum comfortable opening, MCO), occlusal time (OT), and disocclusion time (DT), were recorded and compared at 1 and 3 months.

**Results:**

Significant improvements in pain reduction and mouth opening were observed at both 1 and 3 months of treatment. The T-scan data showed a reduction in both OT and DT after 1 month.

**Conclusion:**

The use of stabilization splints supported by the T-scan system appears to be an effective treatment for TMD, significantly reducing pain and improving mouth opening range (MCO). Although the T-scan system provides valuable insights into occlusal contacts and force distribution, further controlled studies are needed to fully assess its role in optimizing occlusal adjustments during SS treatment.

## Introduction

Temporomandibular disorders (TMD) are a group of conditions that affect the temporomandibular joint (TMJ), masticatory muscles, and associated structures, leading to symptoms such as pain, restricted mouth opening, and impaired function. The prevalence of TMD is substantial, with studies indicating that between 11.3 and 31.1% of the population may experience some form of TMD at some point in their lives, 1 2 with females being more frequently affected. 3 Although occlusion has long been considered a potential factor in TMD, its etiological role remains controversial. A 2017 systematic review by Manfredini et al found only weak associations between TMD and certain occlusal features such as the slide from the retruded contact position to the intercuspal position and the presence of non-working side interferences. These links were not strong enough to confirm a causal relationship. 4 TMD are now understood to result from a complex interplay of biological, psychological, and behavioral influences—including stress, anxiety, and parafunctional habits like bruxism. 1 2 5

Among the treatment options for TMD, stabilization splints (SS) are commonly used as a conservative, non-invasive method. SS, also known as Michigan splints, are designed to improve occlusal stability by redistributing occlusal forces, reducing muscle tension, and preventing joint hyperactivity. 6 Numerous studies have highlighted the effectiveness of SS in reducing pain and improving joint function. A network meta-analysis by Al-Moraissi et al found that hard SS alone significantly reduced post-treatment pain intensity in both arthrogenous and myogenous TMD, with moderate- to low-quality evidence. 7 Similarly, a systematic review by Si-Hui Zhang et al concluded that occlusal splints positively impact mandibular movement and pain reduction in TMD patients. Most included studies reported significant improvements in chronic pain intensity, mouth opening, and TMJ function, supporting the use of splints as a non-invasive treatment approach. 8

Traditional occlusal analysis methods, such as articulating paper, shim stocks, and occlusal waxes, have long been employed to record contact points between the maxillary and mandibular teeth. However, these methods lack the ability to measure the intensity of occlusal forces, which can only be inferred from the size of the marks left on the paper. Studies have shown that relying on the size of articulating paper marks is an unreliable method for assessing occlusal force distribution, as clinicians are generally unable to consistently distinguish between high and low force contacts through visual interpretation alone. 9 Recent advancements in digital occlusal analysis, such as the T-scan system, offer the potential to overcome these limitations. The T-scan system provides real-time digital recordings of occlusal force distribution, including timing, magnitude, and location of the forces, allowing for more accurate data for occlusal adjustment. 10

The effectiveness of the T-scan system in the management of TMD has been supported by several studies. For instance, Thumati and Thumati (2014) demonstrated that reducing disclusion time through T-Scan III-guided occlusal adjustments significantly alleviated symptoms such as temporal headaches, jaw pain, and facial tension in patients with myofascial pain dysfunction syndrome. 15 Similarly, a randomized controlled trial showed that disclusion time reduction therapy guided by T-Scan III resulted in a marked decrease in masticatory muscle hyperactivity and TMD symptoms compared with a placebo control. 16 Although these findings highlight the therapeutic value of T-scan-guided occlusal analysis, studies specifically examining its combination with SS remain limited. One such study by Zhe Li et al found that integrating T-scan with splint therapy improved the precision of occlusal adjustments by providing real-time data on bite timing and force distribution. 11 The T-scan system also enhanced the detection of occlusal asymmetries and helped optimize splint design for more balanced force distribution. These results suggest that combining digital occlusal analysis with splint therapy may improve both functional outcomes and symptom relief in TMD patients. As more studies are conducted at various centers, clinicians will develop a clearer and more objective understanding of the combined effectiveness of SS and the T-scan system.

Thus, the goal of this study is to evaluate the treatment outcomes of TMD using SS supported by the T-scan system. By focusing on both patient characteristics and clinical improvements, including pain reduction and mouth opening range, this study provides insights into the effectiveness of SS in the treatment of TMD in general, as well as the supportive role of the T-scan system in optimizing occlusal adjustments of SS.

## Materials and Methods

### Participants

A total of 36 patients (7 male, 29 female), aged between 18 and 65 years, were included in this study. These patients were selected from those seeking treatment at the Department of Oral and Maxillofacial Surgery, Hanoi Medical University Hospital from December 2023 to October 2024. Patients were diagnosed with TMD based on the Diagnostic Criteria for Temporomandibular Disorders (DC/TMD), 17 a standardized system used in both clinical and research settings. This classification has been incorporated into the American Academy of Orofacial Pain (AAOP) guidelines 14 ensuring consistency in diagnosis and management.

Individuals were excluded if they had systemic diseases such as rheumatoid arthritis or fibromyalgia, unstable general health conditions, psychological disorders that could interfere with treatment compliance, ongoing use of other treatments for TMD, or a history of maxillofacial surgery within the past 6 months.

### Clinical Characteristics

To describe the clinical characteristics of TMD patients, the following parameters were assessed at the baseline prior to treatment: tinnitus, joint dislocation, headache, jaw fatigue, neck and shoulder pain, deviated mouth opening, limited mouth opening, muscle pain, crepitus (clicking, popping), and joint pain. These measurements were not reevaluated post-treatment.

### Treatment Protocol

A total of 36 patients were treated with SS (Michigan-type splints). The splints were custom designed for each patient based on the initial occlusal analysis. The SS were fabricated from hard acrylic and self-curing resin.

Occlusal adjustment for the SS was performed by a single physician, initially using articulating paper to assess the depth and extent of the marks. This step was repeated until there were no noticeable premature contacts and the occlusal force on both sides felt balanced by the patient. After this initial adjustment was verified by the patient, confirming the absence of high spots or interferences, further refinement was made with the T-scan system (Tekscan, Norwood, MA, USA). The patient was seated upright with the occlusal plane parallel to the floor, and the T-scan system was used to measure the asymmetry index of occlusal force (AOF), which indicates the difference in force distribution between the left and right sides of the teeth. After each T-scan measurement, articulating paper was re-applied and using the combined data from both the T-scan system and the paper, any high spots or occlusal interferences were adjusted accordingly. This process was repeated until the patient's occlusion showed no premature contact, and the bilateral force balance was confirmed by the T-scan system, with AOF values less than 10%. No occlusal adjustments were made to the natural teeth.

The patients were instructed to wear the splints primarily at night. The treatment continued for a period of 3 months, with follow-up visits scheduled for 1 and 3 months after starting the treatment.

### Outcome Assessments

- **Pain** : The visual analog scale (VAS) is a 100-mm line used to measure pain intensity. Participants were asked to mark a point on the line to indicate their pain level, where 0 representing no pain and 10 representing the worst pain. VAS scores were recorded at baseline, 1 month, and 3 months.
- **Mouth opening** : The maximum comfortable opening (MCO) was measured in millimeters, determined by the distance between the incisal edges of the maxillary and mandibular incisors, ensuring no pain was caused during the measurement. MCO was measured at baseline, 1 month, and 3 months.
- **Occlusal time (OT)** : OT was measured using the T-scan system on natural teeth before and 1 month after wearing the SS.
- **Disocclusion time (DT)** : DT was recorded using the T-scan system on natural teeth before and 1 month after wearing the SS.

### Statistical Analysis

Data were analyzed using paired *t* -test to compare the changes in OT, and DT at baseline and 1 month. One-way repeated measures ANOVA was used to compare the changes in VAS scores, MCO measurements at baseline, 1 month, and 3 months. A *p* -value of less than 0.05 was considered statistically significant.

## Results

### Demographic Information

Table 1 shows the demographic information of participants. The mean age was 33.1 years (SD = 16), with 80.6% female and 19.4% male. The majority were students (41.7%), followed by office workers (33.3%). Other occupations included farmers (5.6%) and retirees (8.3%).

**Table 1:** Demographic information

Parameters	Number	%
Age, mean (SD)	33.1 (16)	−
Sex		
* Male*	7	19.4
* Female*	29	80.6
Occupation		
* Student*	15	41.7
* Office worker*	12	33.3
* Farmer*	2	5.6
* Retirement*	3	8.3
* Other*	4	11.1

#### Clinical Characteristics

Joint pain was the most common symptom, affecting 86.1%, followed by joint sounds (61.1%), with bilateral sounds being the most frequent. Muscle pain was reported by 44.4% of patients, and neck and shoulder pain by 27.8%. Limited and deviated mouth opening were both present in 33.3% of patients. Other symptoms included jaw fatigue (25%), headache (11.1%), and tinnitus (2.8%) ( Table 2 ).

**Table 2:** Clinical characteristics

Symptoms	Number	Percentage (%)
Tinnitus	1	2.8
Joint dislocation	2	5.6
Headache	4	11.1
Jaw fatigue	9	25
Neck and shoulder pain	10	27.8
Deviated mouth opening	12	33.3
Limited mouth opening	12	33.3
Muscle pain	16	44.4
Joint pain	31	86.1
Mouth opening path		
* Deviated opening*	12	28
* Zigzag opening*	9	17
Joint sound	22	61.1
* Right only*	8	22.2
* Left only*	6	16.7
* Both sides*	8	22.2

#### Clinical Outcomes

The results revealed significant improvements in clinical outcomes over the 3-month treatment period.

*Pain (VAS)* : Muscle pain decreased from a baseline of 4.6 ± 1.2 to 2.2 ± 0.8 at 1 month, and further improved to 1.1 ± 0.9 in 3 months. Joint pain also showed a significant reduction from 4.5 ± 1.2 at baseline to 2.4 ± 0.9 at 1 month, and 1.4 ± 0.6 at 3 months. Both reductions were statistically significant ( *p* < 0.001).

*Mouth opening (MCO)* : The mean maximum comfortable opening (MCO) improved significantly from 32.8 ± 3.6 mm at baseline to 38.3 ± 1.9 mm at 1 month and 41.0 ± 2.3 mm at 3 months, indicating a steady increase in jaw mobility over the course of treatment ( *p* < 0.001).

*OT and DT* : OT decreased from 1.10 ± 0.30 seconds at baseline to 0.80 ± 0.20 seconds at 1 month. DT decreased from 0.90 ± 0.15 seconds at baseline to 0.70 ± 0.11 seconds at 1 month. Both parameters showed significant improvements ( *p* < 0.001) after 1 month of treatment ( Table 3 ).

**Table 3:** Clinical outcomes

Parameters	Baseline	1 month	3 months	*p*
**Pain (VAS)**				
* Muscle pain*	4.6 ± 1.2	2.2 ± 0.8	1.1 ± 0.9	<0.001
* Joint pain*	4.5 ± 1.2	2.4 ± 0.9	1.4 ± 0.6	<0.001
Mouth opening (MCO)	32.8 ± 3.6	38.3 ± 1.9	41.0 ± 2.3	<0.001
OT	1.10 ± 0.30	0.80 ± 0.20	−	<0.001
DT	0.90 ± 0.15	0.70 ± 0.11	−	<0.001

Abbreviations: DT, disocclusion time; MCO, maximum comfortable opening; OT, occlusal time; VAS, visual analog scale.

## Discussion

Although this study used a convenience sampling method, the gender distribution (80.6% female) supports the higher prevalence of TMD in females, which is consistent with findings from other studies. 3 This gender disparity is often associated with behavioral, hormonal, or psychosocial factors, which can exacerbate TMD symptoms, although definitive conclusions have yet to be established. 18 19 In terms of clinical symptoms, joint pain (86.1%) and joint sounds (61.1%) were the most commonly reported in this study. These are often the primary symptoms in TMD. 20 Pain is often the primary reason patients seek medical help, while joint sounds may occur due to changes in condyle morphology, disk displacement, or mechanical disk derangements, even without pain or major functional issues. Additionally, 27.8% of participants reported neck and shoulder pain, highlighting the interconnected nature of TMD and musculoskeletal pain. The presence of pain in the neck and shoulders suggests that TMD and these symptoms may influence each other, contributing to the complexity of the disorder.

The use of SS in our study resulted in a significant reduction in pain and improvement in jaw mobility, confirming their effectiveness in treating TMD. The pain scores, measured by the VAS, dropped noticeably from 4.6 ± 1.2 to 1.1 ± 0.9 after 3 months, averaging 0.3 points per week. The SS treatment also led to an increase in the MCO. The average MCO improved from 32.8 ± 3.6 mm at baseline to 41.0 ± 2.3 mm after 3 months. This relief is primarily due to the splints' ability to set the jaw into a relaxed state, balance the occlusion, and provide support to the TMJ, thereby reducing strain on both the joint and surrounding muscles. By restoring neuromuscular balance, SS help adjust the occlusion and prevent premature tooth contact, which decreases pressure on the TMJ and alleviates muscle tension and joint pain. This process improves the alignment between the jaw and the joint, leading to better pain management and enhanced joint function. 21

Supporting our findings, a 2020 comparative randomized study assessed the impact of SS over a 12-week period in 80 participants with TMJ arthralgia. The results showed significant pain reduction, with VAS pain scores dropping from 6.4 ± 1.5 to 2.0 ± 1.3. 22 Similarly, Bhattacharjee et al conducted a meta-analysis with a minimum 6-month follow-up, comparing SS with other treatments like arthrocentesis in patients with disk displacement without reduction. The analysis showed that SS resulted in moderate pain relief and improved mouth opening, with VAS pain scores improving from 7.5 ± 2.0 to 3.5 ± 1.2. 13 In contrast, a randomized controlled trial by Qvintus et al evaluated SS over 1 year in 80 patients. Significant improvements were reported in pain relief, with reductions in both muscle and joint pain, as well as enhanced jaw mobility and decreased TMJ discomfort at the 12-month follow-up. However, no significant difference was found between the SS group and the control group, which received counseling and exercises. 23 Based on the results of our study and in conjunction with other studies, we believe that SS are an effective treatment option for many patients. However, the necessity for their use may depend on the severity and progression of the condition, as some patients may experience improvement with less invasive treatments or even spontaneously.

In our study, the T-scan system was used to assist in adjusting the occlusion of the SS. Although traditional methods, such as articulating paper and shim stocks, can also provide basic information about occlusal contacts, T-scan offered more precise, real-time data on occlusal contact timing and force distribution, to fine-tune the SS by identifying imbalances and premature contacts on the splint. The real-time and dynamic feedback allows for precise detection of occlusal interferences that are not easily visible with traditional methods. Digital occlusal analysis provides reproducible, quantifiable data, which can support the monitoring and potential refinement of occlusal conditions in patients with myofascial pain. Furthermore, by visually demonstrating occlusal force distribution to patients, T-scan fosters better understanding and compliance, reinforcing its role in both therapeutic intervention and patient education. 24 Compared with the study by Zhe Li et al, 11 which showed significant improvements in occlusal balance (OT, DT, AOF) and functional scores (MCO) after 3 months of T-scan-guided splint adjustment, our findings demonstrated similar clinical improvements after just 1 month of treatment. Although our study lacked a control group, the rapid reduction in VAS scores and enhanced occlusal parameters suggest that digital occlusal refinement can yield meaningful short-term benefits in patients with myofascial TMD.

OT and DT have been linked to increased masticatory muscle activity and excessive loading on the TMJ, contributing to TMD symptoms. 25 Studies indicated that OT should ideally be less than 0.2 seconds, while DT should not exceed 0.4 seconds to maintain balanced occlusal function and minimize joint stress. 11 Monitoring these parameters might provide valuable insights into occlusal dynamics, aiding in the assessment of TMD severity and treatment response. However, caution is warranted when interpreting OT and DT values in the context of TMD. A study by Kuć et al demonstrated that soft tissue mobilization can significantly reduce OT and DT, yet concluded that these parameters cannot be considered as cofactors of the existing TMD—myofascial pain with referral. 12 In our study, after 1 month of using the SS, the OT and DT values reduced but remained relatively high, 0.80 ± 0.20 and 0.70 ± 0.11, respectively, which was expected given that no direct occlusal adjustment was made on the natural teeth. The splint primarily served to alleviate pain and reduce muscle strain, but it did not address the underlying dental issues that contribute to prolonged OT and DT. Although patients reported symptom relief, the continued high OT and DT indicate that the occlusal imbalance was not fully corrected. This suggests that further interventions, such as restorative, selective enameloplasty or orthodontic treatment, may be necessary to optimize occlusal function.

Recent studies have demonstrated the effectiveness of T-scan-guided techniques such as immediate complete anterior guidance development (ICAGD) in reducing myofascial pain and improving occlusal function by shortening DT. 15 16 Although our study focused on splint therapy alone, incorporating ICAGD in future protocols could help delineate the specific contributions of direct enamel adjustment in TMD management. Compared with ICAGD, which uses selective enameloplasty to eliminate posterior interferences, splint-based adjustment is conservative and reversible, modifying only the acrylic surface. Although both methods can relieve symptoms, ICAGD may provide faster and more lasting improvements when DT remains elevated despite splint use. The selection of invasive treatments should be approached with caution. Future comparative studies are needed to clarify which strategy offers the most effective and sustainable occlusal outcomes for TMD patients.

There are several limitations related to the application of the T-scan system in our study. The sensor thickness of 100 microns might impact the accuracy of occlusal measurements, especially when detecting subtle discrepancies. Additionally, the process required detailed patient instruction, which could be time-consuming. The high cost of the T-scan system is another factor, requiring clinicians to balance the benefits of improved occlusal adjustments with financial investment, especially for routine use. These factors should be considered when integrating digital occlusal analysis into standard TMD management protocols.

## Conclusion

The use of SS with the support of T-scan for treating TMD has proven to be effective in improving pain relief and jaw mobility. However, further research with control groups is needed to confirm the distinct role of SS and T-scan in the treatment of TMD, as well as to better understand their individual contributions to improving patient outcomes.
